# Beat-to-beat coronary wave intensity analysis: implications of backward waves originating from microvascular

**DOI:** 10.3389/fcvm.2025.1655193

**Published:** 2025-10-30

**Authors:** Xinzhou Xie, Peng Han, Tiantong Yu, Zixi Huang, Heqiang Lin, Songyun Xie, Bohui Zhang, Shuai Zhao, Che Wang, Chengxiang Li, Yan Chen, Kun Lian

**Affiliations:** ^1^ Department of Information Engineering, School of Electronics and Information, Northwestern Polytechnical University, Xi’an, Shaanxi, China; ^2^ Department of Cardiology, Xijing Hospital, Air Force Military Medical University, Xi’an, Shaanxi, China; ^3^ Department of Cardiology, 981st Hospital of Joint Logistics Support Force, Chengde, Hebei, China; ^4^ Department of Cardiology, Honghui Hospital, Xi’an Jiaotong University, Xi’an, Shannxi, China; ^5^ Department of Cardiology, Air Force Hospital of Western Theater Command, Chengdu, Sichuan, China; ^6^ School of Public Health, Shaanxi University of Chinese Medicine, Xianyang, Shaanxi, China; ^7^ Department of Cardiology, No. 971 Hospital of the PLA Navy, Qingdao, Shandong, China

**Keywords:** coronary wave intensity analysis, coronary physiology, adenosine-induced changes, myocardial-coronary interaction, hemodynamic

## Abstract

**Introduction:**

While coronary wave intensity analysis (cWIA) offers a promising way to assess myocardial and microvascular function by decomposing microvascular-originated backward waves, its clinical utility is currently limited by complex data acquisition and unclear influence of varying hemodynamic factors (adenosine, stenosis and vessel type).

**Methods:**

This study introduces an angiography-based cWIA method and clarifies how those hemodynamic factors impact cWIA parameters. This retrospective study included 124 patients with 125 target vessels, for which beat-to-beat cWIA was successfully performed at rest and during adenosine-induced hyperemia.

**Results:**

Our analysis revealed a strong and significant correlation between cumulative backward compression wave intensity (*cBCW*) and cumulative backward decompression wave intensity (*cBDW*) in both resting (*rho* = 0.846, 95%CI: 0.786 to 0.891, *p* < 0.001) and hyperemic states (*rho* = 0.768, 95%CI: 0.681 to 0.833, *p* < 0.001). Compared to rest, adenosine-induced hyperemia significantly increased *cBCW* (1.88 ± 1.46 ×10^4^ W/m^2^s vs. 2.31 ± 1.74 × 10^4^ W/m^2^s, *p* < 0.001) and peak backward compression wave intensity (*pBCW*) (4.30 ± 4.61 ×10^5^ W/m^2^s^2^ vs. 5.21 ± 4.68 × 10^5^ W/m^2^s^2^, *p* = 0.008), while significantly decreasing peak backward decompression wave intensity (*pBDW*) (5.41 ± 6.06 × 10^5^ W/m^2^s^2^ vs. 3.99 ± 4.64 × 10^5^ W/m^2^s^2^, *p* < 0.001), with no significant effect on *cBDW*. Neither functional stenotic lesions nor vessel type [left anterior descending coronary artery (LAD) or right coronary artery (RCA)] significantly impacted quantitative parameters of the microvascular-originated backward waves.

**Discussion:**

The clinical feasibility of a convenient cWIA method was validated, and the impact of various hemodynamic factors on quantitative parameters of cWIA were analyzed, providing strong support for the clinical application of cWIA.

## Introduction

The interplay between the coronary arteries and the myocardium (cardiac-coronary coupling) endows coronary blood flow with distinct pulsatile characteristics (1, 2). While established hemodynamic assessment techniques, such as fractional flow reserve (FFR) and coronary flow reserve (CFR), furnish valuable insights into myocardial perfusion, they often neglect the wealth of information inherent in the pulsatile nature of coronary blood flow (3–5). Coronary wave intensity analysis (cWIA) is a technique that analyzes the time derivatives of coronary pressure and flow velocity waveforms (6). By separating forward waves, which are generated by the aorta, from backward waves, which result from microvascular, cWIA quantifies the energy of these waves (7). Among these waves, the backward decompression wave (BDW) and the backward compression wave (BCW) are considered to potentially reflect pathological changes (8, 9). BDW is closely associated with left ventricular diastolic function, correlating with relevant indices, and can predict capillary density changes and the risk of cardiac allograft vasculopathy in heart transplant patients (8, 10, 11). In hypertrophic cardiomyopathy, BDW's reduction and its correlation with left ventricular septal thickness help evaluate the impact of left ventricular hypertrophy (12). Furthermore, existing literature suggests that BDW may also serve as an indicator for assessing coronary microvascular dysfunction (13, 14). BCW amplitude, which correlates with myocardial contractility indices, is useful for assessing the relationship between coronary hemodynamics and myocardial function (11, 15). In aortic stenosis, BCW decreases with increasing heart rate, impairing coronary physiological reserve, but this reverses after TAVR, highlighting its potential for assessing aortic stenosis' impact on coronary flow and intervention efficacy (16).

While cWIA holds considerable promise for coronary physiology research, its clinical translation is hindered by several obstacles. Firstly, cWIA necessitates the simultaneous acquisition of pulsatile coronary pressure and blood flow velocity. While pulsatile pressure measurement within the coronary arteries is well-established in clinical practice, pulsatile blood flow velocity measurement remains a significant challenge (17). Currently, Doppler-based techniques are the primary method for this purpose (18). However, the inherent motion artifacts associated with continuous cardiac activity introduce substantial difficulties in obtaining stable Doppler signals (17). Furthermore, the requirement for simultaneous acquisition of pulsatile pressure and blood flow velocity increases the complexity and cost of the procedure. Secondly, the BDW and BCW originate from coronary microvasculature. This microvasculature, serving as the primary regulator of coronary blood flow, exhibits significant variations in vascular tone across different physiological states (with or without functional stenosis, resting or hyperemic states) (19). However, the precise influence of these hemodynamic variations on the quantification of the characteristic waveforms remains poorly understood. This uncertainty raises concerns regarding the reliability of coronary disease diagnosis and prognostic evaluations based on BDW and BCW quantification.

The primary objective of this study is to advance the clinical application of WIA in coronary disease assessment. Firstly, we implement a novel coronary angiography-based method for the simultaneousness calculation of coronary blood flow, leveraging fractional flow reserve (FFR) (20). This approach, based on a novel hemodynamic model, utilizes routine angiographic images and FFR measurements to derive the synchronous blood flow velocity data required for WIA, thereby significantly streamlining the data acquisition process (20). This study will validate, for the first time, the feasibility of applying this method to coronary WIA. Secondly, this paper endeavors to examine the impact of diverse hemodynamic factors, including adenosine-induced hyperemic states, coronary functional stenosis, and vascular territory (left anterior descending coronary artery (LAD) or right coronary artery (RCA)), on the quantitative parameters of BDW and BCW, gaining a deeper understanding of the physiological significance of BDW and BCW.

## Materials and methods

### Study population

Between June 2022 and June 2024, patients aged 18 years or older who were referred for diagnostic coronary angiography and fractional flow reserve (FFR) assessment at the Department of Cardiology, Xijing Hospital, Fourth Military Medical University, due to stable or unstable angina were screened for enrollment. Written informed consent was obtained in all patients and the study was approved by the research ethics board (Medical Ethics Committee of the First Affiliated Hospital of the Fourth Military Medical University, KY20222192-F-1). Exclusion criteria were as follows: (1) acute myocardial infarction (ST-elevation or non-ST-elevation); (2) allergy to iodine-based contrast agents; (3) significant bleeding risk or coagulation disorders (e.g., malignancy); (4) presence of anemia, infectious diseases, or severe pulmonary diseases; (5) severe ventricular arrhythmias or hemodynamic instability. Angiographic exclusion criteria included suboptimal image quality or severe vascular overlap that precluded accurate assessment.

### Pulsatile pressure and flow velocity measurement

#### Pulsatile pressure measurement

Pulsatile pressure data was gathered using conventional FFR measurement techniques with the QUANTEIN and PRESSUREWIRE systems (Abbott Global Health Care, USA). Before insertion, the devices underwent a three-time *in vitro* zeroing process, which included the catheter chamber pressure channel, aortic pressure, and pressure guidewire pressure. Following this, the aortic and guidewire pressures were equilibrated to a shared baseline. The pressure guidewire was then carefully guided across the target lesion, stopping 2–3 cm distal to it. Once the pressure waveform stabilized, maximal hyperemia was initiated through a continuous intracoronary infusion of adenosine triphosphate (ATP). Finally, both the pressure waveforms and FFR values were meticulously recorded.

#### Pulsatile flow velocity calculation

**Figure 1:**
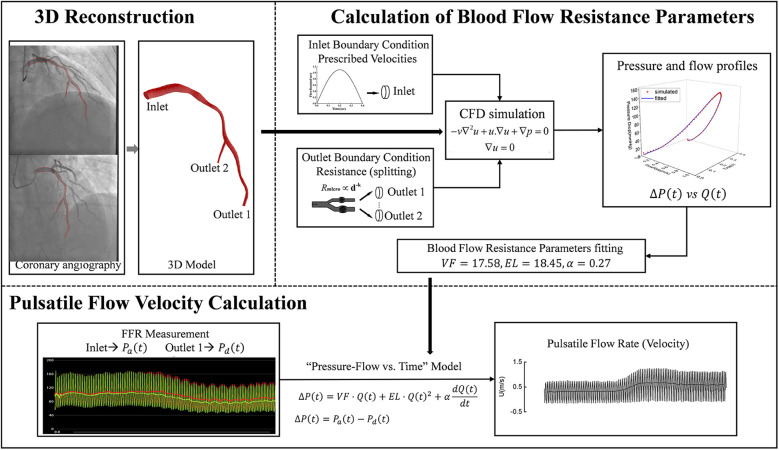
Pulsatile blood flow velocity calculation workflow. First, a 3D model of the target vessel was reconstructed from two coronary angiography images. Next, CFD was used to simulate pulsatile pressure and blood flow within the 3D model under predefined boundary conditions, and the vessel resistance model parameters were fitted based on these simulation results. Finally, pulsatile blood flow and velocity were calculated by combining the FFR-acquired inlet and outlet pressure waveforms with the fitted vessel resistance model parameters. CFD: computational fluid dynamics; VF, viscous friction; EL, expansion loss; α, inertia term coefficient; ΔP(t), pressure drop across the epicardial coronary artery; P_a_(t), proximal pressure; P_a_(t), distal pressure; Q(t), flow rate; U, flow velocity.

To calculate pulsatile flow velocity using coronary angiography images and FFR, the following sequential steps are implemented (as illustrated in Figure 1):

1. 3D Reconstruction: Multi-angle angiography images, acquired at 15 frames per second from various perspectives, were utilized for 3D model reconstruction. We reconstructed the 3D centerlines of the target coronary vessel and its major branches (defined as those with a diameter greater than 1 mm and a length exceeding 2 cm) using a point-cloud-based method (21) from two angiographic projections, which were separated by at least 25°. Following this, circular luminal contours were fitted along all identified vascular pathways, creating the complete 3D anatomical model of the coronary artery;
2. Calculation of Blood Flow Resistance Parameters: Based on the reconstructed 3D anatomical model, the flow resistance parameters of the epicardial coronary artery were calculated. The pressure drop across the epicardial coronary artery would be approximated by the equation (20):
  $$\Delta P(t)=VF\cdot Q(t)+EL\cdot Q{\left(t\right)}^{2}+\alpha \frac{dQ(t)}{dt}$$

Where, *VF* represented viscous friction, *EL* denoted expansion loss, and *α* was the inertia term coefficient for pulsatile flow. These parameters, which are determined by the anatomical structure of the epicardial coronary artery, were derived using a previously proposed computational method (20). The fundamental principle of this method is as follows: we first use computational fluid dynamics (CFD) simulations to generate pulsatile blood flow and pressure drop data, and then employ a fitting method to inversely derive the model parameters from this generated data. Detailed procedures of the computational method are provided in the Supplementary Material;

3. Pulsatile Flow Velocity Calculation: Pulsatile pressure drops were derived from the measured pressure values of FFR, and were refined by using the Savitzky-Golay convolution method (22). Then, with the pressure drops and the pre-calculated flow resistance parameters (*VF*, *EL*, and *α*), the pulsatile blood flow rate *Q*(*t*) and flow velocity *U*(*t*) [dividing *Q*(*t*) by the cross-sectional area of the distal end] were calculated by using the finite-difference method (20).

### Coronary wave-intensity analysis

cWIA was performed using MATLAB R2021a (MathWorks, Natick, MA, USA) to quantitatively assess energy transfer characteristics within coronary arteries. Wave speed was calculated via the single-point method, using the formula $c\;=\;\surd (\Delta P^{2}/\Delta U^{2})$ (23). A key methodological choice was to use the wave speed from the resting state for the analysis of both resting and hyperemic conditions. This decision addresses the documented issue of the single-point method underestimating wave speed during hyperemia (24), thereby ensuring a more reliable and consistent comparison between the two states. The net wave intensity (WI) was derived from arterial blood pressure and blood flow velocity signals, applying the formula WI=(dP/dt)⋅(dU/dt) (6). The resulting wave-intensity profile was automatically characterized by compression (*dP*/*dt* > 0) vs. decompression (*dP*/*dt* < 0), and was subsequently separated into its forward and backward components (6, 25). The variables of interest in this study were the peak wave energy, cumulative wave intensity, and the proportion of cumulative wave intensity of the forward compression wave (FCW), forward decompression wave (FDW), BCW and BDW under resting and hyperemic conditions. The cumulative wave intensity of each wave was calculated by measuring the area under the curve (12). The proportion of cumulative wave intensity was calculated by expressing the cumulative wave intensity of an individual wave as a percentage of the total cumulative wave intensity in the cardiac cycle (12). For reliable computational results, three consecutive cardiac cycles exhibiting stable pressure waveforms (a time period with no significant variation, defined as a variation rate of no more than 5%—in cycle duration, systolic pressure, and diastolic pressure) were chosen from both resting and hyperemic conditions. The average of the cWIA quantitative parameters derived from these three cycles were subsequently employed for statistical analysis.

### Statistical analysis

All statistical analyses were carried out using GraphPad Prism 9.5.0 (GraphPad Software, La Jolla, CA, USA). Continuous variables are presented as their mean ± standard deviation. For paired measurements, differences were assessed using the paired-samples *t*-test if the differences were normally distributed. If the differences were not normally distributed, the paired-sample Wilcoxon signed-rank test was used. For group comparisons, the independent-samples *t*-test was applied if the data for each group were normally distributed and variances were equal. Otherwise, the Mann–Whitney *U* test was performed. Spearman's correlation coefficient was applied to evaluate linear relationships between variables. A *p*-value of <0.05 was considered to indicate statistical significance.

## Results

A total of 137 patients with simultaneously recorded angiographic images and fractional flow reserve (FFR) data were retrospectively reviewed. Of these, 8 patients were excluded due to missing angiographic views or severe vessel overlap precluding 3D model reconstruction, and 5 patients were excluded due to incomplete FFR data. Consequently, 124 patients with 125 target vessels were ultimately included in the final analysis. Out of the 125 target vessels, 94 (75.20%) in LAD, 8 (6.40%) in left circumflex coronary artery (LCX), 23 (18.40%) in RCA and 24 vessels (19.20%) with a positive FFR (≤0.80). Further baseline variables for the included patients were described in Table 1.

**Table 1:** Baseline characteristics.

Variables	*n* = 125
Age (Year)	62.31 ± 9.87
Males (male, %)	98 (78.40%)
BMI (kg/m^2^)	24.42 ± 2.55
SBP (mmHg)	131.80 ± 18.10
DBP (mmHg)	76.34 ± 11.32
Heart rate (bpm)	76.04 ± 9.22
Hospitalization days (Day)	5.26 ± 2.28
History of smoking (%)	53 (42.40%)
History of drinking (%)	25 (20.00%)
Hypertension (%)	77 (61.60%)
Diabetes (%)	53 (42.40%)
Family history of cardiovascular disease (%)	4 (3.20%)
ALT (U/L)	27.52 ± 14.56
AST (U/L)	23.40 ± 6.64
Creatinine (μmoI/L)	76.97 ± 21.10
TC (mmol/L)	3.21 ± 0.83
TG (mmol/L)	1.68 ± 0.84
LDL-C (mmol/L)	1.54 ± 0.74
HDL-C (mmol/L)	1.21 ± 0.39
cTnI (μg/L)	0.01 ± 0.01
NT-proBNP (ng/L)	253.00 ± 568.90
NYHA functional class	
Ⅰ	54 (43.20%)
Ⅱ	71 (56.80%)
Ⅲ	0 (0.00%)
Ⅳ	0 (0.00%)
LVEF(%)	57.56 ± 4.07
Aspirin (%)	90 (72.00%)
Clopidogrel (%)	58 (46.40%)
Ticagrelor (%)	48 (38.40%)
Statins (%)	125 (100.00%)
Proton pump inhibitor (%)	74 (59.20%)
ACEI/ARB(%)	35 (28.00%)
Beta blockers (%)	104 (83.20%)
Cardiotonic diuretic (%)	0 (0.00%)

BMI, body mass index; SBP, systolic blood pressure; DBP, diastolic blood pressure; ALT, alanine transaminase; AST, aspartate transaminase; FBG, fasting blood glucose; TC, total cholestrol; TG, triglyceride; HDL-C, high density lipoprotein cholestrol; LDL-C, low density lipoprotein cholestrol; NT-proBNP, N-terminal pro-brain natriureticpeptide; cTnI, cardiac troponin I; LVEF, left ventricular.

**Figure 2:**
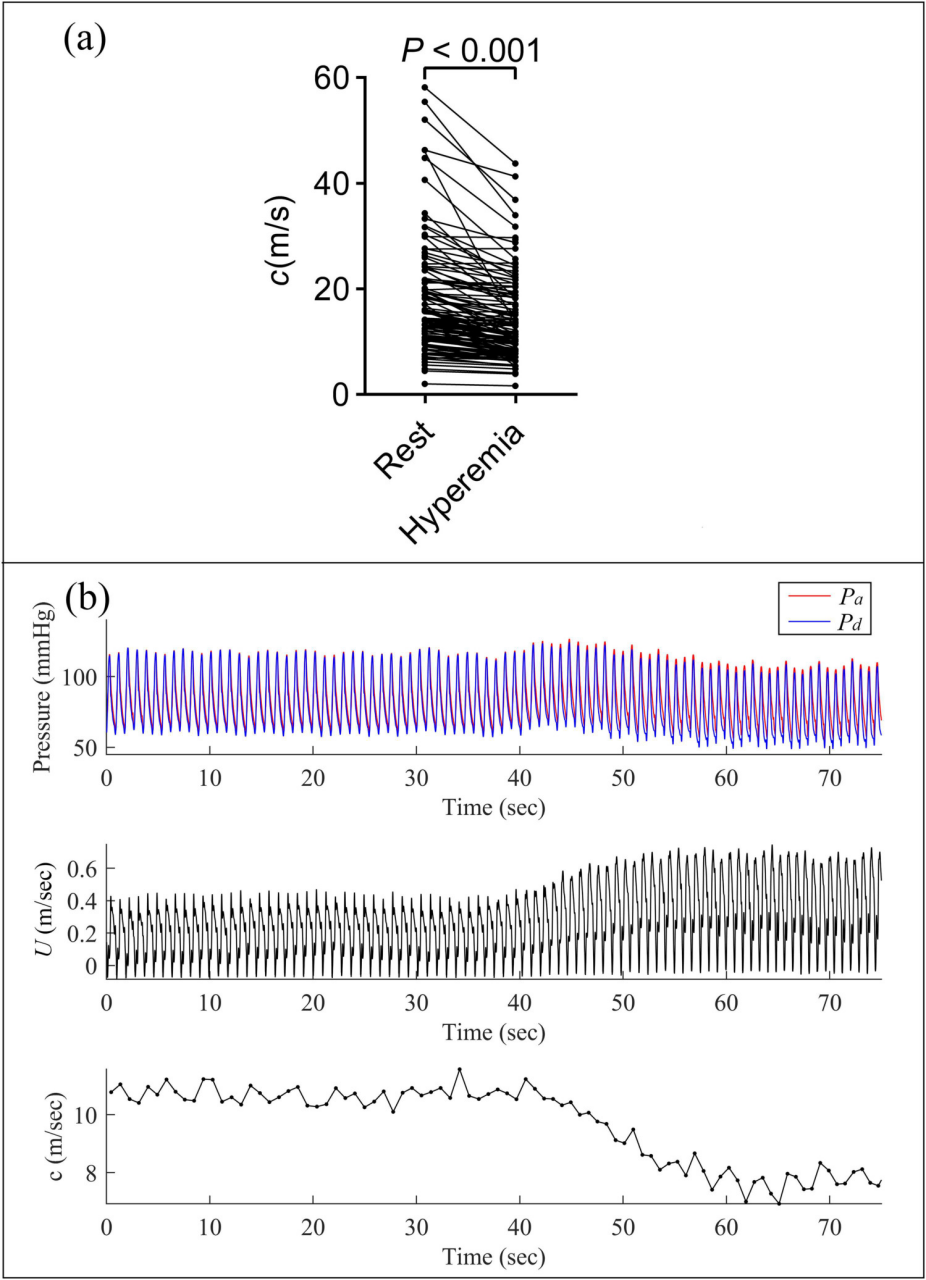
Comparison of pulse wave velocity in resting and hyperemic states. **(a)** Paired *t*-test showed a significant difference in pulse wave velocity between resting and hyperemic states; **(b)** continuous measurements of pressure, blood flow velocity, and pulse wave velocity (calculated using the single-point method) in the left anterior descending (LAD) artery of a 78-year-old female patient. Note the decrease in pulse wave velocity concurrent with increased blood flow velocity following adenosine-induced microvascular dilation.

Figure 2 showed the changes in pressure, blood flow velocity, and pulse wave velocity (calculated by the single-point method) continuously acquired in the LAD of a 78-year-old female patient before and after adenosine infusion. Following adenosine-induced microvascular dilation and increased blood flow velocity, the pulse wave velocity calculated by the single-point method also decreased accordingly. The coronary wave speed was, on average, 19.65 ± 17.27 m/s at rest, which was significantly higher than the average wave velocity of 14.75 ± 10.03 m/s during hyperemia (*p* < 0.001).

As shown in Table 2, the total cumulative wave intensity during hyperemia was slightly higher than at rest, but this difference was not statistically significant (5.32 ± 4.35 × 10^4 ^W/m^2^s vs. 5.70 ± 4.09 × 10^4^ W/m^2^s, *p* = 0.118). In both resting and hyperemic states, BCW and BDW, originating from the microcirculation, were dominant. Adenosine-induced hyperemia significantly increased the energy proportions of BCW (36.52 ± 8.68% vs. 40.63 ± 10.55%, *p* < 0.001), whereas the energy proportions of BDW showed no significant difference between resting and hyperemic conditions (40.22 ± 9.43% vs. 39.36 ± 10.60%, *p* = 0.372).

**Table 2:** Impact of adenosine-induced hyperemia on cWIA wave energy proportions.

Total cumulative wave intensity (×10^4^ W/m^2^s)		Rest	Hyperemia	*P* value
		5.32 ± 4.35	5.70 ± 4.09	0.118
Proportion of cumulative wave intensity (%)	FCW	15.20 ± 8.40	13.59 ± 9.30	0.002
	FDW	8.06 ± 4.02	6.43 ± 4.42	<0.001
	BCW	36.52 ± 8.68	40.63 ± 10.55	<0.001
	BDW	40.22 ± 9.43	39.36 ± 10.60	0.372

Values are means ± SE; FCW, forward compression wave; FDW, forward decompression wave; BCW, backward compression wave; BDW, backward decompression wave.

**Figure 3:**
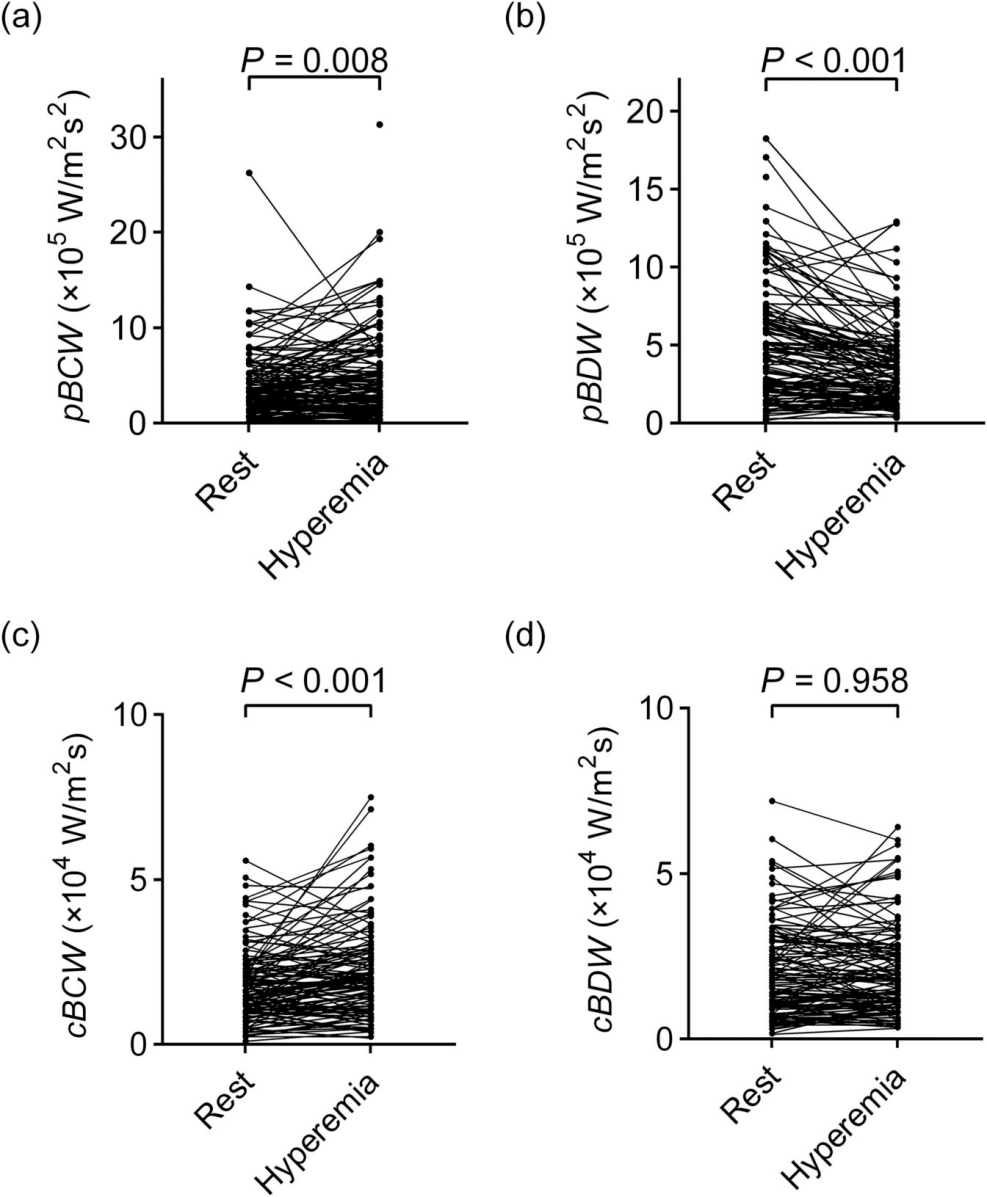
Comparison of quantitative parameters of microvascular-originated backward waves between resting and hyperemic states. **(a)** *pBCW*; **(b)** *pBDW*; **(c)** *cBCW*; **(d)** *cBDW*. *pBCW*, peak backward compression wave intensity; *pBDW*, peak backward decompression wave intensity; *cBCW*, cumulative backward compression wave intensity; *cBDW*, cumulative backward decompression wave intensity.

**Figure 4:**
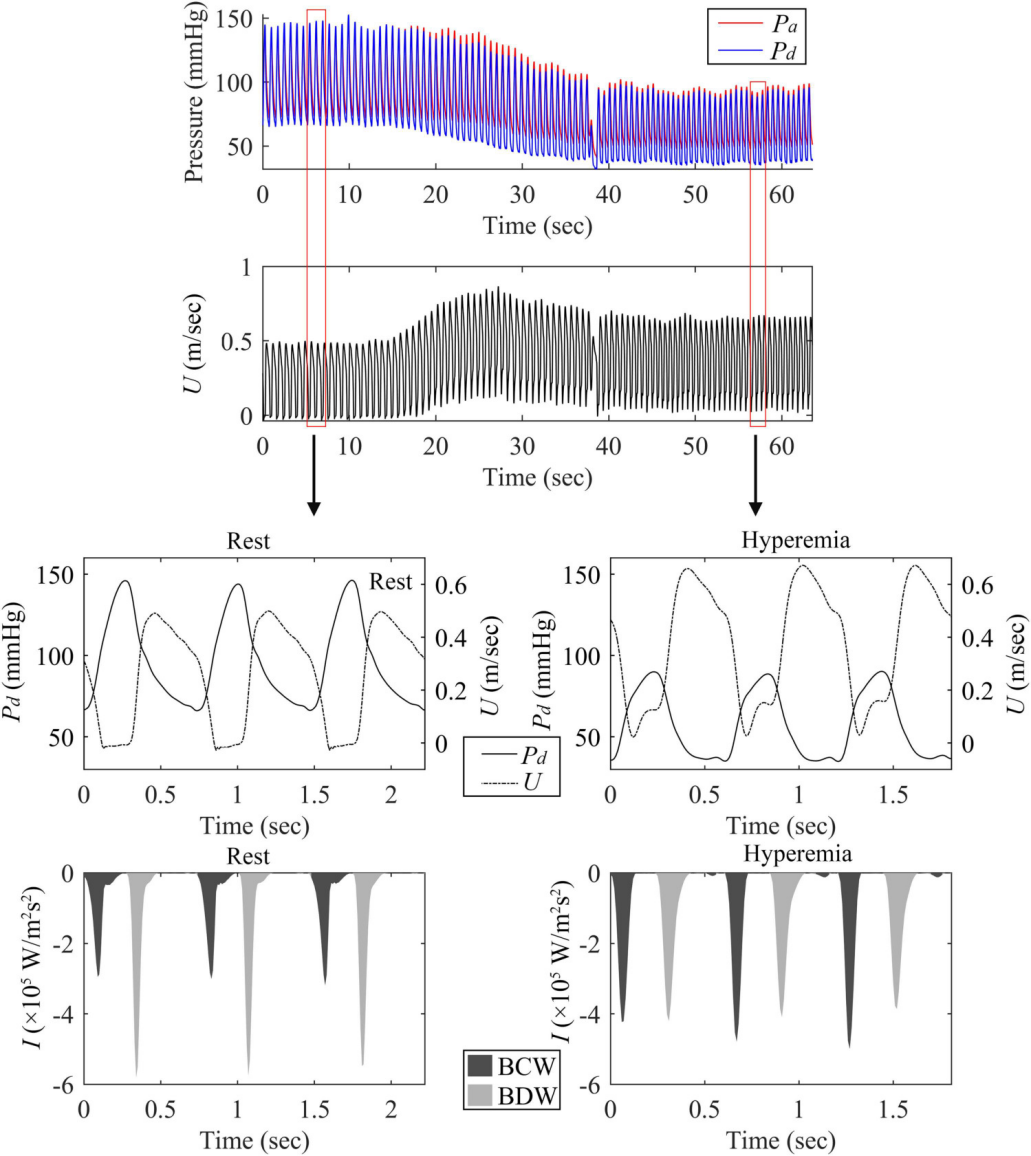
Comparison of BCW and BDW in resting and hyperemic states. This figure shows measurements from the LAD artery of a 69-year-old male patient with a negative FFR of 0.84. The panels display: (top) continuously acquired pulsatile pressure and blood flow velocity; (bottom left) Resting state pressure, blood flow velocity, and backward wave forms; (bottom right) Hyperemic state pressure, blood flow velocity, and backward wave forms.

As depicted in Figure 3; Table 3, adenosine-induced hyperemia significantly increased both the peak BCW intensity (*pBCW*) (4.30 ± 4.61 × 10^5 ^W/m^2^s^2^ vs. 5.21 ± 4.68 × 10^5 ^W/m^2^s^2^, *p* = 0.008) and cumulative BCW intensity (*cBCW*) (1.88 ± 1.46 × 10^4 ^W/m^2^s vs. 2.31 ± 1.74 × 10^4 ^W/m^2^s, *p* < 0.001). Conversely, the peak BDW intensity (*pBDW*) significantly decreased during hyperemia (5.41 ± 6.06 × 10^5 ^W/m^2^s^2^ vs. 3.99 ± 4.64 × 10^5 ^W/m^2^s^2^, *p* < 0.001), while the cumulative BDW intensity (*cBDW*) showed no significant difference between resting and hyperemic states (2.22 ± 2.05 × 10^4 ^W/m^2^s vs. 2.22 ± 1.76 × 10^4 ^W/m^2^s, *p* = 0.958). Figure 4 presented a typical case illustrating the complete cWIA waveform, demonstrating that the *pBCW* was notably higher during hyperemia, whereas the BDW waveform during hyperemia exhibits a lower peak and a longer duration.

**Table 3:** Impact of adenosine-induced hyperemia on quantitative parameters of microvascular-originated backward waves.

Variables	Rest	Hyperemia	*P* value		Rest	Hyperemia	*P* value
*pBCW* (×10^5^ W/m^2^s^2^)	4.30 ± 4.61	5.21 ± 4.68	0.008	*cBCW* (×10^4^ W/m^2^s)	1.88 ± 1.46	2.31 ± 1.74	<0.001
*pBDW* (×10^5^ W/m^2^s^2^)	5.41 ± 6.06	3.99 ± 4.64	<0.001	*cBDW* (×10^4^ W/m^2^s)	2.22 ± 2.05	2.22 ± 1.76	0.958

Values are means ± SE; *pBCW*, peak backward compression wave intensity; *pBDW*, peak backward decompression wave intensity; *cBCW*, cumulative backward compression wave intensity; *cBDW*, cumulative backward decompression wave intensity.

As showed in Table 4, regardless of whether it was at rest or during hyperemia, the coronary wave velocity in the FFR-positive group was slightly higher than that in the FFR-negative group, but the difference was not significant (at rest: 21.64 ± 18.68 m/s vs. 19.17 ± 16.98 m/s, *p* = 0.532; during hyperemia: 16.42 ± 13.91 m/s vs. 14.35 ± 8.91 m/s, *p* = 0.367). As indicated in Table 5, functional stenosis of epicardial arteries had no significant impact on the quantified parameters of microvascular-originated waves. Given the limited number of LCX cases, we confined our analysis to the differences in statistical parameters between LAD and RCA. As shown in Table 6, there were no significant differences in the quantified parameters of both BCW and BDW, for either peak or cumulative wave intensity, between these two vessel types.

**Table 4:** Impact of functional stenosis of epicardial arteries on pulse wave speed.

Condition	Pulse wave speed (*c*) (m/s)		
	FFR ≤ 0.8 (*n* = 24 )	FFR > 0.8 (*n* = 101)	*P* value
Rest	21.64 ± 18.68	19.17 ± 16.98	0.532
Hyperemia	16.42 ± 13.91	14.35 ± 8.91	0.367

Values are means ± SE.

**Table 5:** Impact of functional stenosis of epicardial arteries on quantitative parameters of microvascular-originated backward waves.

Variables		FFR ≤ 0.8 (*n* = 24)	FFR > 0.8 (*n* = 101)	*P* value
Rest	*pBCW* (×10^5^ W/m^2^s^2^)	3.29 ± 2.59	4.53 ± 4.95	0.239
	*pBDW *(×10^5^ W/m^2^s^2^)	5.87 ± 4.33	5.30 ± 6.41	0.678
	*cBCW* (×10^4^ W/m^2^s)	1.45 ± 0.83	1.99 ± 1.56	0.108
	*cBDW* (×10^4^ W/m^2^s)	2.13 ± 1.30	2.24 ± 2.20	0.823
Hyperemia	*pBCW* (×10^5^ W/m^2^s^2^)	5.73 ± 4.70	5.09 ± 4.70	0.551
	*pBDW* (×10^5^ W/m^2^s^2^)	4.84 ± 3.54	3.79 ± 4.85	0.320
	*cBCW* (×10^4^ W/m^2^s)	2.63 ± 1.46	2.23 ± 1.80	0.310
	*cBDW* (×10^4^ W/m^2^s)	2.73 ± 1.68	2.10 ± 1.77	0.116

Values are means ± SE; *pBCW*, peak backward compression wave intensity; *pBDW*, peak backward decompression wave intensity; *cBCW*, cumulative backward compression wave intensity; *cBDW*, cumulative backward decompression wave intensity.

**Table 6:** Differences in quantitative parameters of microvascular-originated backward waves between LAD and RCA.

Variables		LAD (*n* = 94 )	RCA (*n* = 23)	*P* value
Rest	*pBCW* (×10^5^ W/m^2^s^2^)	4.29 ± 5.00	4.91 ± 3.45	0.575
	*pBDW *(×10^5^ W/m^2^s^2^)	5.50 ± 6.59	5.88 ± 4.43	0.792
	*cBCW *(×10^4^ W/m^2^s)	1.94 ± 1.59	1.94 ± 1.59	0.866
	*cBDW *(×10^4^ W/m^2^s)	2.30 ± 2.26	2.20 ± 1.32	0.838
Hyperemia	*pBCW *(×10^5^ W/m^2^s^2^)	5.22 ± 4.72	5.39 ± 4.82	0.879
	*pBDW* (×10^5^ W/m^2^s^2^)	4.21 ± 5.13	3.40 ± 2.93	0.466
	*cBCW* (×10^4^ W/m^2^s)	2.36 ± 1.77	2.29 ± 1.87	0.859
	*cBDW *(×10^4^ W/m^2^s)	2.36 ± 1.91	1.81 ± 1.31	0.194

Values are means ± SE; *pBCW*, peak backward compression wave intensity; *pBDW*, peak backward decompression wave intensity; *cBCW*, cumulative backward compression wave intensity; *cBDW*, cumulative backward decompression wave intensity.

**Figure 5:**
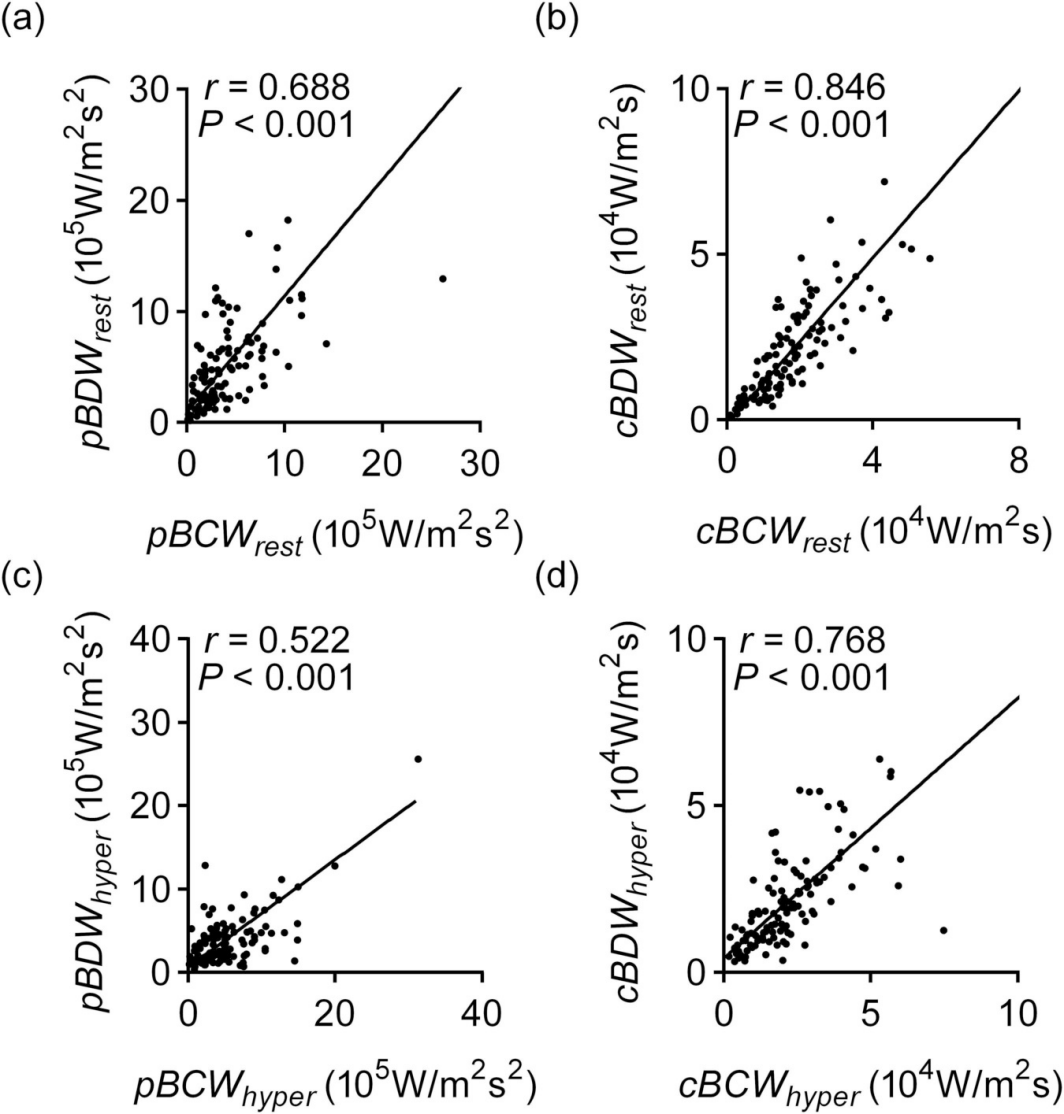
Correlation of BCW and BDW quantitative parameters in resting and hyperemic states. **(a)** Correlation of *pBCW* and *pBDW* at rest; **(a)** correlation of *cBCW* and *cBDW* at rest; **(c)** correlation of *pBCW* and *pBDW* during hyperemic; **(d)** correlation of *cBCW* and *cBDW* during hyperemic. *pBCW_rest_*, peak backward compression wave intensity at rest; *pBDW_rest_*, peak backward decompression wave intensity at rest; *cBCW_rest_*, cumulative backward compression wave intensity at rest; *cBDW_rest_*, cumulative backward decompression wave intensity at rest; *pBCW_hyper_*, peak backward compression wave intensity during hyperemic; *pBDW_hyper_*, peak backward decompression wave intensity during hyperemic; *cBCW_hyper_*, cumulative backward compression wave intensity during hyperemic; *cBDW_hyper_*, cumulative backward decompression wave intensity during hyperemic.

As shown in Figure 5, *cBCW* and *cBDW* exhibited a very strong correlation, reaching 0.846 (95%CI: 0.786–0.891, *p* < 0.001) during the resting state. This correlation decreased to 0.768 (95%CI: 0.681–0.833, *p* < 0.001) under hyperemic conditions. Similarly, *pBCW* and *pBDW* also showed a strong correlation in the resting state (*rho* = 0.688, 95%CI: 0.580–0.773, *p* < 0.001), with a decrease in correlation observed during hyperemia (rho = 0.522, 95%CI: 0.377–0.642, *p* < 0.001).

## Discussion

### Comparison to traditional cWIA analysis

Traditional cWIA was performed based on blood flow signals measured by Doppler ultrasound technology. Due to the relatively low signal-to-noise ratio of these signals, it was necessary to ensemble the blood flow signals from multiple cycles with reference to the synchronously acquired ECG signals (26). However, as shown in Figure 2, there were certain variations in the blood flow and pressure signals of consecutive cardiac cycles, which would have a certain impact on the cWIA analysis. In this paper, the blood flow velocity signals used for cWIA analysis were calculated based on FFR and blood flow resistance model. Beat-to-beat cWIA analysis was achieved, without the need for multi-cycle signal superposition and ensemble. This method has certain advantages over traditional methods in terms of operational complexity and analysis principles. The *pBCW* and *pBDW* values calculated by our method in the resting state were on the order of 10^5 ^W/m^2^s^2^, and the magnitudes of *cBCW* and *cBDW* were on the order of 10^4^ W/m^2^s, which aligns with the ranges reported in previous literature (11, 16, 27).

### Coronary pulse wave speed

Coronary pulse wave speed serves as an indicator of coronary arterial wall elasticity and provides valuable insights for the diagnosis and prognosis of coronary artery disease (28, 29). In contrast to the two-point measurement technique, the single-point method offers notable advantages in the coronary arteries. The conventional approach relies on quantifying the transit time of the pressure wave between two spatially defined points (24). However, due to the limited length of coronary arteries and the challenge of obtaining adequate time intervals, this method is impractical in this vascular bed (23, 24). The single-point method, which involves simultaneous measurement of pressure and flow velocity, circumvents these limitations. While the single-point method provides reliable estimates of coronary wave speed at rest, it has been documented to underestimate wave speed by approximately 40% during hyperemia (24). Our results aligned with the aforementioned reports in that adenosine-induced hyperemia significantly reduced the pulse wave velocity calculated by the single-point method. However, the average reduction observed in this study was approximately 25%, which is slightly less than that reported in previous literature. Given this observation, the cWIA analysis for the hyperemic state in this paper still utilized the pulse wave velocity calculated under resting conditions. Furthermore, comparisons between FFR-negative and FFR-positive cohorts demonstrated no significant differences in wave speed, either at rest or during hyperemia.

### Dominated waves and their relationship

The interplay between the myocardium and the coronary arteries gives the coronary arteries special pulsating nature. The BCW and BDW, which start in the microvasculature, hold most of the energy of the pulse wave, both at rest and during hyperemia. Earlier research had suggested that the BCW was related to ventricular systolic function, while the BDW more reflected ventricular diastolic function (10–12, 15). However, there hasn't been much research looking at how these two waves relate to each other. Our results indicated that BCW and BDW exhibit a very strong correlation in the resting state, for both peak and cumulative wave intensity. This strong correlation suggests that, despite appearing at different times during the cardiac cycle, the two waves may be more intrinsically linked than previously thought. Therefore, the common practice of independently considering them as distinct indicators of systolic or diastolic function, as done in the past, is debatable. Our findings point to the need for a more integrated understanding of their relationship in the assessment of coronary physiology.

### Impact of adenosine-induced hyperemia

Only a limited number of studies had investigated the impact of adenosine on quantitative cWIA parameters. Claridge et al. reported significant increases in both *cBCW* and *cBDW* from measurements in 36 vessels across 8 subjects following adenosine administration (11). Similarly, DeMarchi et al. observed a significant rise in *cBDW* induced by adenosine (26). However, our results presented a contrasting picture: adenosine-induced hyperemia led to a significant increase in *pBCW* and *cBCW*, but a significant decrease in *pBDW*, with no significant effect on *cBDW*. This discrepancy might stem from methodological differences: DeMarchi et al. required alignment and reconstruction of 30-s continuously acquired raw pressure and blood flow velocity data based on ECG (26). Given adenosine's very short half-life [typically around 20 s (30)], this extended data segment may have inadvertently included phases where adenosine's effect was diminishing. In contrast, our study analyzed data from independent cardiac cycles, ensuring greater temporal accuracy of the data. Furthermore, our results were consistent with the observed cWIA waveform changes: the BDW waveform exhibited a lower peak and a longer duration during hyperemia. The hemodynamic changes induced by adenosine are relatively complex: in addition to the significant increase in diastolic blood flow velocity caused by microvascular dilation, a notable decrease in perfusion pressure can also be clearly observed, which may have an impact on the myocardial-coronary interaction. Our results suggest that the interaction between myocardial relaxation and coronary blood flow become less intense but more prolonged under hyperemic conditions. In addition, it can be seen from the experimental results that the quantitative parameters of cWIA are greatly affected by adenosine, which suggests that changes in the baseline hemodynamic state have a significant impact on the quantitative analysis of cWIA. When attempting to investigate the relationship between the absolute values of these parameters and various physiological or pathological changes, special attention must be paid to the corresponding baseline hemodynamic state.

### Impact of flow-limited stenosis

Conflicting reports existed regarding the influence of functional stenosis on quantitative cWIA parameters. Narayan et al. compared cWIA parameters in 17 patients before and after percutaneous coronary intervention (PCI) and found significant increases in *cBDW* post-PCI (31). In contrast, DeMarchi et al. reported no significant correlation between BDW quantitative parameters and the presence of stenotic lesions (26). Our results indicated that while there were some differences in cWIA wave quantitative parameters between the FFR-positive and FFR-negative groups, these differences did not reach statistical significance. We therefore hypothesized that despite functional stenosis leading to a reduction in distal perfusion pressure and associated blood flow, its impact on the interaction between the myocardium and coronary blood flow was limited.

### Differences between LAD and RCA

It is generally believed that the interaction between the myocardium and coronary blood flow is significantly weaker in the RCA compared to the LAD, primarily due to the substantially lower pressures in the right ventricle (25). However, our results showed no significant differences in the quantitative parameters of the microvascular-originated waves between these two vessel types, regardless of whether in a resting or hyperemic state, or during systole or diastole. We further analyzed the pressure and flow velocity waveforms collected from RCA cases. Among 23 RCA vessels, 21 exhibited instances where *P_d_* was greater than *P_a_* during systole, leading to clearly observable retrograde blood flow. Our findings point to the possibility that the myocardial-coronary interaction also significantly influences the pulsatile flow pattern in the RCA, a hypothesis that warrants further investigation.

### Limitations

Several limitations of this study warrant consideration. First, the blood flow calculation method employed in this study was applicable only to patients with epicardial obstructive lesions who underwent FFR examination. This inherent selection criterion might introduce a degree of bias into the patient cohort. Second, individual variability in blood viscosity and rheology—a well-recognized challenge across CFD-based methodologies—may introduce deviations in our calculation results, which might be a potential source of systematic error. Third, the number of FFR-positive cases and RCA cases included in this study was relatively small. Despite our current statistical findings of no significant impact on the cWIA quantification results, future research is warranted to expand the sample size for further validation. Finally, due to existing constraints, this study was unable to investigate the clinical diagnostic value of cWIA quantitative parameters in coronary artery disease, a crucial area for future research.

## Conclusion

This study utilized a low cost approach, leveraging angiographic images and FFR pressure data, to calculate pulsatile coronary blood flow velocity and subsequently perform cWIA. This method enables beat-to-beat cWIA analysis and simplifies the data acquisition process. We validated the feasibility of applying this method for clinical cWIA analysis. Building on this, we found a strong and significant correlation between BCW and BDW quantitative parameters. Further analysis revealed that adenosine-induced hyperemia significantly enhanced *pBCW* and *cBCW* while significantly decreasing *pBDW*, with no significant impact on *cBDW*. Furthermore, functional stenosis of epicardial arteries showed no significant effect on BCW and BDW quantitative parameters. Finally, our analysis did not observe significant differences in the quantitative parameters of microvascular-originated waves between different coronary branches (LAD and RCA).

## Data Availability

The raw data supporting the conclusions of this article will be made available by the authors, without undue reservation.
