# Supplementary material for: Beat-to-beat coronary wave intensity analysis: implications of backward waves originating from microvascular
Source: Front Cardiovasc Med. 2025 Oct 30;12:1655193. doi: 10.3389/fcvm.2025.1655193 (PMC12611823; doi:10.3389/fcvm.2025.1655193)
Supplement: Supplementary file 1 [file Datasheet1.docx]

**Supplementary Material**

The method for pulsatile flow velocity calculation adopted in this study is founded on a series of foundational studies that have continuously refined and validated the core methodology. Its development can be traced through three key stages: firstly, the theoretical framework for modeling pressure drop and blood flow in stenotic vessels using empirical formulas was initially established in the 1970s [1]; secondly, the researchers' previous work utilized detailed numerical simulations to confirm the validity of these empirical formulas even with vessel bifurcations, further developing a method to calculate mean blood flow via FFR and angiographic images and preliminarily validating its clinical value against SPECT data [2, 3, 4]; thirdly, their most recent work advanced the method by adding an inertial term to the model, enabling the calculation of pulsatile blood flow and more complex analysis [5].

The pressure drop across the epicardial coronary artery would be approximated:

$$\Delta P\left( t \right)=VF\cdot Q\left( t \right)+EL\cdot{Q\left( t \right)}^{2}+\alpha\frac{dQ(t)}{dt}$$

The three core blood flow resistance parameters—**viscous friction (VF)**, **expansion loss (EL)**, and **inertia term coefficient (α)**—are critical for describing the pressure drop characteristics of pulsatile blood flow in epicardial coronary arteries. Their physical meanings and roles in the model are defined as follows:

***VF***: Represents the frictional resistance caused by blood viscosity and vessel wall roughness, which is linearly related to the flow rate (*Q*(*t*)) and dominates the pressure drop at low flow velocities.

***EL***: Accounts for the energy loss due to flow separation and recirculation, which exhibits a quadratic relationship with *Q*(*t*) and becomes more significant at high flow velocities.

***α***: Reflects the inertial effect of pulsatile blood flow, which is proportional to the rate of change of flow rate (*dQ*(*t*)/*dt*) and is crucial for capturing the dynamic pressure drop during cardiac cycle transitions.

These parameters, which are determined by the anatomical structure of the epicardial coronary artery, were derived using a previously proposed computational method[5]. The specific calculation process of the model parameters is as follows:

1. Pulsatile CFD simulations were performed on the reconstructed 3D coronary artery model (by using the open-source software package SimVascular) to obtain pulsatile pressure drops and blood flow rates that could be used for model parameter extraction. Key simulation settings included:

Blood properties: Dynamic viscosity = 4.0 cP, density = 1060 kg/m³.​

Boundary conditions: Pulsatile inlet flow (sine-wave waveform, 60–90 bpm) and Windkessel-modeled outlet resistance (based on Murray’s Law) to mimic microvascular resistance.​

Data extraction: After 3 cycles of simulation (to eliminate transients), the last cycle’s pulsatile flow rate and pressure drop were extracted as the reference dataset for parameter fitting.

1. Parameters Fitting: The simulated pulse blood flow rate and pulse blood pressure drop at the target vessel are derived into MATLAB R2019a (The MathWorks, Inc., Natick, MA, USA), these data points are combined in the three-dimensional coordinate system of flow-pressure-time to obtain the curve of the fluid dynamics, then VF, EL and α are estimated by iterative least squares estimation method of nonlinear regression.

In essence, we first use CFD simulations to generate a set of precise "reference data." We then use a mathematical fitting method to inversely derive the model parameters from this data, rather than calculating them directly. This approach ensures that the model parameters accurately reflect the hemodynamic characteristics of the vessel.

**References**

[1] Young DF, Tsai FY. Flow characteristics in models of arterial stenoses — I. Steady flow. Journal of Biomechanics. 1973;6(4):395-410.

[2] Xie X, Zheng M, Wen D, et al. A new CFD based non-invasive method for functional diagnosis of coronary stenosis. BioMed Eng OnLine. 2018;17(1):36.

[3] Li W, Lian K, Chen Y, et al. Computing intracoronary blood flow rate under incomplete boundary conditions: Combing coronary anatomy and fractional flow reserve. Med Eng Phys. 2023;111:103942.

[4] Xie X, Li N, Yu T, et al. Validation of a Novel Computational Fluid Dynamics Based Method for Assessing Intracoronary Flow Combining Coronary Angiography and Fractional Flow Reserve. Catheter Cardiovasc Interv. 2025; 105(7):1695-1706.

[5] Pu W, Chen Y, Zhao S, et al. Computing pulsatile blood flow of coronary artery under incomplete boundary conditions. Med Eng Phys. 2024;130:104193.
